# Patient- or person-centred practice in medicine? – A review of concepts

**DOI:** 10.4102/phcfm.v9i1.1455

**Published:** 2017-10-19

**Authors:** Jakobus M. Louw, Tessa S. Marcus, Johannes F.M. Hugo

**Affiliations:** 1 Department of Family Medicine, School of Medicine, Faculty of Health Sciences, University of Pretoria, South Africa

## Abstract

**Background:**

Person-centred practice in medicine may provide solutions to several pressing problems in health care, including the cost of services, poor outcomes in chronic care and the rise in litigation. It is also an ethical imperative in itself. However, patient- or person-centred care is not well researched partly because of a lack of conceptual and definitional clarity.

**Aim:**

The aim of this review was to analyse essential elements, ethical principles, logic and the practical application of person-centred practice described in clinician- and researcher-defined conceptual frameworks, terms and practices.

**Methods:**

A search of review articles on patient- and person-centred care or medicine was conducted using Medline and Google Scholar. Secondary searches were conducted using references and citations from selected articles.

**Results:**

Five conceptual frameworks were identified in terms of their practical application of the ethical principles of beneficence, autonomy and justice. They converge around a few central ideas such as having a holistic perspective of patients and their illness experience, a therapeutic alliance between the patient and clinician as well as respectful, enabling collaboration with the patient.

**Conclusions:**

Terminological differences appear to owe more to disciplinary origins than to substantive meaning. Beneficence needs to be balanced by and practised through respect for patient autonomy. Core ideas in existing conceptual frameworks of patient or person centredness can guide teaching and research. Considering the value and ethical imperative of person-centred practice, training institutions should train health care students and practitioners in its precepts.

## Introduction

The concept of patient or person centredness has gained prominence internationally and received official support in the health care systems of several countries including the USA, UK, Germany and Australia.^1^

The concept of patient-centred care (PCC) developed mostly in the discipline of family medicine, whereas the concept of person-centred medicine was defined and mostly developed in psychiatry. The difference in emphasis suggested by these terms can be attributed to their origins. In the practice of the former, the idea of PCC is used to shift the focus of the consultation away from the clinician and associated medical practices to the patient and their expectations, fears, feelings, etc.^2^ In the person-centred medicine movement, advocates focus on both the person of the patient and the person of the clinician as well as their contexts – the society they live in and the health system they function in.^3,4^

Person-centred practice has a number of actual or potential benefits. Even though evidence is still regarded as insufficient by some, it can improve patient health outcomes.^5,6,7,8,9^ It may also reduce the workload (and cost) of health care services^10,11^ by avoiding services and procedures that patients do not want or need.^11,12^ Person-centred care increases patient satisfaction,^13^ reduces complaints against health care professionals and leads to fewer malpractice lawsuits.^14,15^ Person-centred practice is also important for the development of patient capability.^16^ Entwistle and Watt^16^ contend that person-centred care should be pursued for its own intrinsic value as it is an ethical requirement that clinicians treat patients as persons with significance. They argue that ‘the ways others treat us enable us (or not) to exhibit the characteristics – as well as to experience the social status – of persons as ethically significant beings.’^16^

While there is no universally agreed-upon definition of person-centred practice,^1^ an abundance of terms are used in the medical and health care literature to describe its intent, including person-centred medicine, person-centred care, patient centredness, individualised medicine, personalised medicine, family-centred medicine, patient-centric medicine, patient-centric care, etc.^1,13,17^ The multiplicity of terms and the absence of a singular definition reflect the complexity as well as the state of flux of person centredness as a practice. The profusion of descriptions can also be attributed to the roots and specific applications of person-centred practice in a variety of disciplines including family medicine, psychiatry, nursing, dentistry, physiotherapy and others.

This article is an analytic review of clinician-defined conceptual frameworks as well as researcher-defined terms and practices of person-centred practitioners. Frameworks, terms and practices were identified and then assessed in terms of their underlying ethical principles, logical construction and practical application in order to ascertain similarities and differences.

In terms of ethical values, the review focuses primarily on beneficence and autonomy, with some references to respect, non-maleficence and justice. These values are described by the Health Professions Council of South Africa (HPCSA) as follows:^18^

- **Respect for persons:** Health care practitioners should respect patients as persons, and acknowledge their intrinsic worth, dignity, and sense of value.
- **Best interests or well-being:**
  ■ **Non-maleficence:** Health care practitioners should not harm or act against the best interests of patients, even when the interests of the latter conflict with their own self-interest.
  ■ **Beneficence:** Health care practitioners should act in the best interests of patients even when the interests of the latter conflict with their own personal self-interest.
- **Autonomy:** Health care practitioners should honour the right of patients to self-determination or to make their own informed choices, and to live their lives by their own beliefs, values and preferences.
- **Justice:** Health care practitioners should treat all individuals and groups in an impartial, fair and just manner.

## Methods

Searches were conducted on the databases of Ovid Medline®, Pubmed and Google Scholar for English language articles published between 2000 and 2015. The search terms were ‘patient centredness’, ‘patient centred’, ‘person centredness’, ‘person centred’, ‘model’, ‘concept’, ‘definition’ and ‘framework’.

Searches in the three databases rendered approximately 4500 articles of possible relevance to the understanding of person- or patient-centred practice with sufficient variety in terms of sources and content. To build a clear understanding of the concept and for the sake of feasibility, the search was further refined to include review articles that described a framework, model or conceptual definition of person or patient centredness. It yielded approximately 900 articles.

Through a review of titles, articles with a disease or age-specific focus (e.g. stroke or the elderly) were excluded. Similarly, articles describing person or patient centredness in terms of a specific service such as rehabilitation or nursing homes were excluded. Secondary searches were then conducted in the references and citations of the most relevant articles. The criteria for inclusion were the potential for application in medical practice, ethical implications and logical clarity. Through these processes the eight sources discussed below were selected.

### Ethical considerations

Ethical clearance was obtained from the University of Pretoria Faculty of Health Sciences Research Ethics Committee (Ethics reference no.:128/2013).

## Review findings

Six of the sources describe five frameworks for person- or patient-centred medicine in generalist primary care. In addition, two sources reviewing the dimensions, themes and behaviours of person-centred practice are discussed. These eight sources come from a range of disciplines in health care (family medicine,^19,20,21^ psychiatry,^3^ medical psychology^1^ and nursing^13^) and from a health policy perspective.^12,24^ The frameworks have been described over a period of two decades (1995^22^ to 2014^12,19^).

The following are the five frameworks:

- the six (later four) interactive components of the patient-centred clinical method described by Stewart^20^ and Stewart et al.^19^
- the five key dimensions of patient centredness described by Mead and Bower^21^
- definitions and descriptions by Miles and Mezzich^3^ in their model of person-centred medicine
- four defining attributes of person-centred practice described by Morgan and Yoder^13^
- four principles of person-centred care described by Collins.^12^

The first four frameworks are applicable to the medical consultation where a clinician meets with a patient to find solutions for health-related problems. The fifth framework relates more to the health care system, how it is accessed and how it interfaces with patients. In the analysis that follows, they are discussed and compared in terms of their practical application, logical construction and ethical implications.

### The six interactive components of the patient-centred clinical method

**FIGURE 1:**
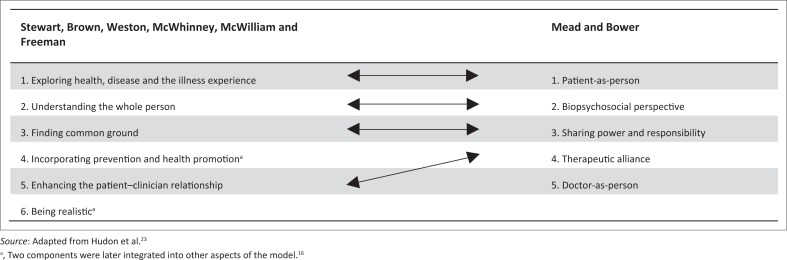
Patient-centred care: A comparison of the Stewart et al.^19^ and Mead and Bower^21^ frameworks based on Hudon et al.^23^

The first framework for consideration is the patient-centred clinical method described by Stewart et al.^19^ Stewart^20^ describes patient centredness as ‘the middle way’ where there is a balance (equally valued) between the individual and the community, science and art, analysis and synthesis, and technology and wisdom. In pursuing this balance, she contends that clinicians will regain their capacity for love and spirituality.^20^ This patient-centred or integrated clinical method comprises six interactive components (Figure 1).

The first three interactive components follow the common sequence of medical consultations from understanding the patient fully through the medical history and examination to finding common ground on the assessment (diagnosis) and management plan. In the third edition of their book, the authors integrated prevention and health promotion into the other interactive components.^19^ Being realistic is no longer described as a component but rather as a comment on the implementation of this clinical method.

This clinical method is focused on fully and deeply understanding the patient for the benefit of the patient. Thus, it is the practical manifestation of the ethical principle of beneficence. In the first component, the focus is on understanding the patient’s experience of the illness. The second component builds on this by exploring the person and the context of the patient. This flows well into ‘finding common ground’ (component three): the patient and the clinician coming to one mutually acceptable understanding of both the problem and the plan to address it. On this common ground the patient–clinician relationship can be built (fifth component) through sharing of power and responsibility.

In two of the interactive components, the ethical value of autonomy (of the patient) is inferred. Finding common ground requires the patient to contribute towards a mutual understanding of the problem and of the way forward. In enhancing the patient–clinician relationship, Stewart et al.^19^ refer to the sharing of power. However, it is not clear how much autonomy is offered to the patient to pursue unique, customised options of assessment and management. Incorporation of ‘prevention and health promotion’^22^ (previously component four), for instance, is the clinician’s agenda and, thus, is more a manifestation of beneficence and less of autonomy. In some scenarios, attempts to prevent disease without proper patient involvement in decision-making can cause greater harm than good (e.g. screening for prostate cancer). This breaks the principle of non-maleficence. Thus, the lack of emphasis on patient autonomy is a weakness in this framework.

The ‘being realistic’ comment (previously the sixth component)^19,22^ is unique to this framework. It will aid the practical implementation of any framework of care. It reminds clinicians that the implementation of the first five, very important, interactive components will often be limited by lack of time and other resources. It can also inspire innovative planning to overcome these limitations. Being realistic requires cooperation and collaboration. Through well-organised teamwork, more time is available for building good patient–clinician relationships. A deep relationship with a patient cannot be established in one consultation; being realistic means ensuring continuity of care to allow for the relationship to grow over time. Being realistic can also refer to the ethical principle of justice. The use of resources to the benefit of one patient should not be to the detriment of other patients.

Incorporating prevention and health promotion implies that the patient is capable of learning and changing behaviour. However, if the clinician lectures the patient and does not give the patient autonomy to choose actions, ask questions and contribute to the plan, learning will be limited and the application of new knowledge unlikely.

### The five key dimensions of patient centredness

The framework of Mead and Bower,^21^ analysed next, describes five key dimensions of patient centredness, four of which correlate with the interactive components described by Stewart et al.^19^ as Figure 1 shows.

The first two of the five key dimensions are very similar to the first two components of the framework described by Stewart et al.^19^ They indicate the importance of knowing the patient comprehensively to help the patient comprehensively (beneficence).

The next dimension described by Mead and Bower^21^ is that of sharing power and responsibility. The authors describe how the power of the medical expert conflicts with the patient’s autonomy as a lay person. Reduction of this power imbalance, they argue, requires that the doctor respects patient autonomy and confers decision-making power on the patient as a shared responsibility.

When it comes to the power and responsibility dimension, it is important to note the use of the term ‘sharing’ by Mead and Bower.^21^ The clinician, as medical expert, still carries responsibility and decision-making power as to abdicate these would be unfair and in conflict with the ethical value of justice.

The dimension of the therapeutic alliance in this framework has a strong focus on beneficence. The clinician and the patient form an alliance against suffering and ill health for the benefit of the patient.

The fifth dimension in the framework by Mead and Bower^21^ reminds the clinician to be self-aware. The clinician’s emotional responses and biases influence patient healing and this is not always with positive effect. The clinician’s self-awareness should guide the application of their own person to the benefit of the patient.

### Descriptions of person-centred medicine

In their framework, Miles and Mezzich^3^ describe person-centred medicine as ‘the rational integration’ of the thinking behind two social movements in medical care, namely PCC and evidence-based medicine (EBM). In so doing, they seem to reinvent or redefine person-centred medicine as new or ‘emergent’ when actually the concept and terminology of person-centred medicine predates EBM by several decades and, as discussed in this review, there is no clear differentiation between the concepts of person-centred care and PCC.

According to Miles and Mezzich,^3^ person-centred medicine is:

a move away from impersonal, fragmented and decontextualised systems of healthcare towards personalised, integrated and contextualised models of clinical practice, so that affordable biomedical and technological advance can be delivered to patients within a humanistic framework of care which recognises the importance of applying science in a manner which respects the patient as a person and takes full account of his [*or her*] values, preferences, stories, cultural context, fears, worries and hopes and which thus recognises and responds to his [*or her*] emotional, spiritual and social necessities in addition to his [*or her*] physical needs.^3^

This definition is more like a description with definitional elements that include the system of health care delivery – ‘models of clinical practice’ – and the provision of health care to the individual ‘within a humanistic framework of care’. In it, the ethical principle of beneficence is prominent as the authors seek to incorporate everything that is good and valued in medicine, including the best interest of the patient, into person-centred medicine. It can also be understood as a response to the question of how the health care provider and the health care system can provide the best possible service to each patient.

Also, although Miles and Mezzich^3^ refer to respect for the patient as a person and for the patient’s preferences, they fail to emphasise patient autonomy or describe how the patient should be involved in collaboration and shared decision-making with the health care provider. Their definition is therefore insufficient to guide enquiry or practice in the field.

Like Miles and Mezzich,^3^ The Health Foundation^17^ in the United Kingdom also refers to a person-centred health *system*. The Health Foundation describes this as a health system that ‘supports people to make informed decisions about, and to successfully manage, their own health and care, [to be] able to make informed decisions and choose when to invite others to act on their behalf.’^17^ Thus, the health care service should ‘work in partnership to deliver care responsive to people’s individual abilities, preferences, lifestyles and goals’.^17^ They then define person-centred care as ‘a philosophy that sees patients as equal partners in planning, developing and assessing care to make sure it is most appropriate for their needs’.^17^ This description gives prominence to individual autonomy, but little is said about providing the best quality of care. It lacks a focus on beneficence. The Health Foundation provides evidence of the benefits of self-management support, but without the appropriate checks it may result in patients harming themselves. This is against the ethical principle of non-maleficence. Collaboration with a caring, competent health care professional should reduce this risk.

### Four principles and four defining attributes of person-centred practice

**FIGURE 2:**
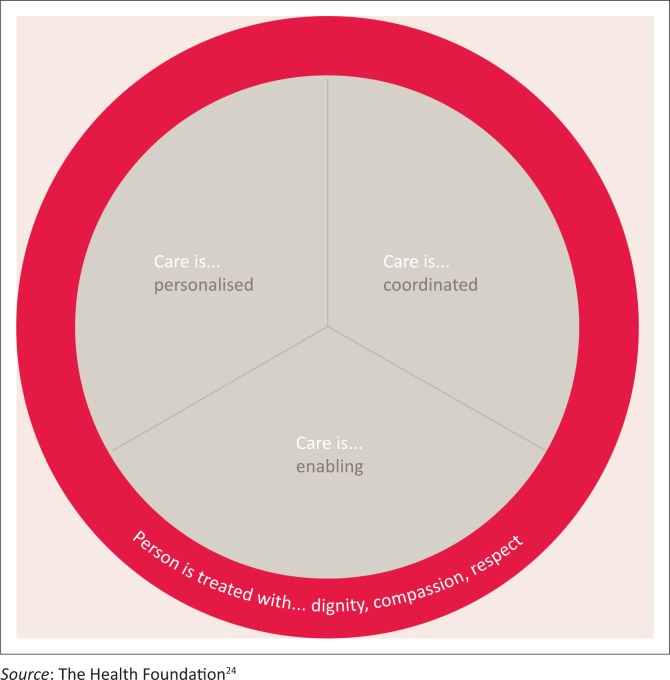
Collins’s four principles of person-centred practice.^12,24^

In the fourth framework, Collins’s^12^ person-centred practice is guided by four principles, namely personalised, coordinated, enabling and compassionate (Figure 2) practice, which supports self-management, shared decision-making and collaborative care and planning.^12,24^

Similarly, Morgan and Yoder^13^ describe what they call defining attributes of person-centred care – namely holistic, individualised, respectful and empowering.

**FIGURE 3:**
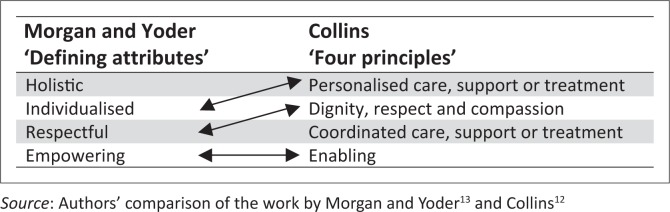
Person-centred care: A comparison of Morgan and Yoder’s^13^ ‘defining attributes’ and Collins’s^12,24^ ‘four principles’.

As Figure 3 demonstrates, three of their ‘defining attributes’ are interchangeable with three of Collins’s four principles.

Both frameworks can be applied to person-centred practice at an organisational level and to some extent at an interpersonal level in medical consultations. Both contain elements of beneficence. In the framework of Morgan and Yoder,^13^ beneficence is articulated as holistic, individualised, respectful care, while in Collins’s^12^ framework it is expressed as personalised care with dignity, respect and compassion. And, unlike the definition by Miles and Mezzich,^3^ both emphasise patient autonomy, describing person-centred care as empowering and enabling.

## Discussion

The terms ‘person centredness’ and ‘patient centredness’ are often used interchangeably in the medical literature.^25^ Although the frameworks were developed in different disciplines, many of their concepts overlap or are similar. In terms of researcher understanding and interpretation of person-centred care, a review of the empirical literature by De Silva^17^ generated a list of 19 subcomponent themes and 19 behaviours associated with person-centred care (Table 1). In a systematic review of PCC, Scholl et al.^1^ integrated more than 400 definitions into one model. They defined 15 dimensions of PCC and categorised them into four principles, five enablers and six activities (Table 2). These lists demonstrate the diversity in descriptions of the concept with some authors (like clinicians) placing more emphasis on certain dimensions, subcomponent themes or behaviours than on others, with many authors only making reference to a few. The result is a multiplicity of definitions and the absence of a single definition that researchers investigating person-centred practice agree on.

**TABLE 1:** Subcomponent themes and behaviours of person-centred care–related research.

Subcomponent themes	Behaviours
1. Activation	1. Advocating
2. Choice	2. Assessing needs^a^
3. Compassion	3. Assessing family needs^a^
4. Continuity	4. Communicating
5. Control	5. Coordinated care
6. Dignity	6. Enablement^b^
7. Empathy^a^	7. Engagement^b^
8. Empowerment	8. Goal planning^b^
9. Health literacy	9. Individual budgets
10. Holism	10. Individual care plans
11. Independence	11. Information provision
12. Individuality	12. Listening^a^
13. Integration	13. Participation
14. Involvement^b^	14. Physical environment
15. Partnership^b^	15. Recognising values
16. Privacy^a^	16. Self-care support^b^
17. Respect	17. Shared decision-making^b^
18. Rights	18. Support^b^
19. Trust	19. Transitions

*Source*: Adapted from De Silva^17^

a , specific to facilitation;

b , specific to collaboration.

**TABLE 2:** Dimensions of patient-centred care by Scholl et al.^1^

Principles	Enablers	Activities
1. Essential characteristics of the clinician	1. Clinician–patient communication	1. Patient information
2. Clinician–patient relationship	2. Integration of medical and non-medical care	2. Patient involvement in care
3. Patient as a unique person	3. Teamwork and teambuilding	3. Involvement of family and friends
4. Biopsychosocial perspective	4. Access to care	4. Patient empowerment
-	5. Coordination and continuity of care	5. Physical support
-	-	6. Emotional support

*Source*: Scholl et al^1^

There appears to be a tension between beneficence and autonomy. While some frameworks have a strong emphasis on beneficence to the detriment of autonomy, others emphasise autonomy without ensuring beneficence.

In terms of ethical principles, Miles and Mezzich^3^ and Stewart et al.^26^ place more emphasis on beneficence and less on patient autonomy. Morgan and Yoder,^13^ Collins^12^ and The Health Foundation^17^ seem to promote patient autonomy more. The model described by Mead and Bower^21^ seems more balanced.

When beneficence is applied without involvement of the patient, it can restrict patient autonomy. However, respect for patient autonomy can also be a catalyst for the activation of the beneficence intended by the clinician, because a patient who is involved in customising a decision is more likely to adhere to the beneficial treatments jointly decided upon in a therapeutic alliance.

In patient-centred medicine, a more symmetrical power relationship between the patient and the clinician may prevent patients being submitted to unnecessary or even harmful treatment by clinicians who may stand to gain financially or otherwise from such treatment. Thus, when the clinician lacks beneficence, patient centeredness to support autonomy can prevent maleficence. Paradoxically, this apparent benefit is absent in the literature on person centredness. Perhaps the notion of a clinician who pursues own self-interest above the patient’s best interest is the elephant in the room that nobody dares to name.

Close collaboration in the therapeutic alliance or the patient–clinician relationship enables strong effective therapeutic interventions and it has therapeutic value in itself. Positive expectations in this alliance lead to positive outcomes.^27^ As Rogers^28^ described, the unconditional positive regard, congruence and empathy within the alliance effect improvement in a patient’s condition. Balint^29^ described the doctor as the medication: ‘the drug, doctor’.

This alliance can be skewed if one of the parties invests more into it and the other lacks commitment and investment or if one party yields significantly more power than the other. Sharing of power is essential to prevent abuse of any of the parties in the alliance. An example is where the patient may manipulate a clinician to prescribe medications such as antibiotics or habit forming medication to the detriment of the patient. The patient uses the emotional investment in the alliance to convince the clinician to act against clinical knowledge. Alternatively, there is the example of a surgeon convincing a patient within their alliance to have a back operation that is without significant health benefit but has financial gain for the medical industry. These examples are in conflict with the ethical value of non-maleficence.

Entwistle and Watt^16^ reasoned that models of person-centred care should be based on capability rather than on patient autonomy. In the conceptual frameworks analysed, capability is referred to by Collins,^12^ Morgan and Yoder^13^ and The Health Foundation^17^ in the terms empowerment and enabling. Capability seems to be absent in the other frameworks.

Where a clinician focuses only on respecting the patient’s autonomy, the clinician may provide all the information regarding the patient’s disease and treatment options and leave the patient to decide without helping the patient or building a relationship with the patient. For some patients this may be scary and unhelpful.^16^ However, in a capability approach to person-centred care, the clinician will work with the patient to increase their ability to confidently make health care decisions. In response, Frank^30^ also contends that respect for patient autonomy includes respecting a patient’s right to choose not to make a decision or to allow or not allow a clinician or someone else to decide for them. This shows the value of both the autonomy and capability approaches in implementing person-centred practice.

The term ‘patient centred’ is often used to refer to the clinical consultation and the direct relationship between the patient and the health care provider. As we have seen, it has also been used to refer to a health care system or even health care policy. In terms of the patient–health care provider interaction, the notion of ‘patient’ indicates that the parties are meeting with a specific purpose, namely the health of the patient, and in this interaction they are neither equals nor do they come with equal expectations.

By replacing the notion of ‘patient’ with that of ‘person’, it reminds medicine of its epicentre: the person of the patient as well as the people who are significant to that person, such as family, caregivers and friends.^3^ The term ‘person’ is also suggestive of a sense of equality with the health care provider.^16^

Miles and Mezzich^3^ contend that the conceptual difference lies in where the obligation to care is placed. Person-centred care is not simply about providing care to patients on their own terms. Rather, care is the result of shared decision-making between two people, the person of the patient and the person of the clinician ‘focussed on the patient’s best interests, in a caring atmosphere, within a relationship of engagement, responsibility and trust’.^3^

In practice, however, all these meanings are also found in patient-centred frameworks, conceptually and in method.^21,31^ As Figure 1 shows, Stewart et al.^19^ include prevention and health promotion (typically the doctor’s initiative) and Mead and Bower^21^ include the dimension of ‘doctor-as-person’^21^ in PCC. Thus, seeing ‘the patient as person’ and ‘the doctor as person’ are fundamental tenets of both person-centred medicine and PCC. It can be concluded, therefore, that there is little in conceptual intent that differentiates the person centred and patient centred debate.^32^

Defining person-centred practice remains complex … so many authors, so many definitions. Multiple terms are used in the literature to describe this concept. However, an analysis of descriptions of the elements, dimensions, attributes, components, etc., of person centredness reveals that they converge around a few core concepts.

Person-centred medicine attempts to achieve the same ideals promoted by PCC and the biopsychosocial approach that Paul Tournier^23,33^ (Medicine of the Person) and others advocate. As to whether it is possible to provide whole medicine by whole practitioners for whole people or even to put the person of the patient at the centre of the clinical encounter^34^ remains an empirical question as does the ideal of integrating all that is good into general primary health care.

## Conclusions

Medicine is practised on the basis of ethical values within a contract between society and health care providers. Person-centred practice can be viewed as the practical manifestation of these values, focusing particularly on the importance of patient autonomy and the practice of beneficence. One of the core values in the practice of medicine is beneficence – to do good, to do the best for each patient. Beneficence needs to be balanced by and practised through respect for the patient’s autonomy. To do this requires collaborative practice. The call to collaborative person-centred practice is actually a call to respect the autonomy of each person while also building their capacity for autonomy as a capability.

Notwithstanding the multiplicity of definitions and terms used to describe person- or patient-centred practice, conceptually there is notional convergence around a few core principles and dimensions of practice. These include a holistic perspective of patients and their illness experience, a therapeutic alliance between the patient and clinician as well as respectful, enabling collaboration with the patient. Executed as well-intended, skilful collaboration, such practice can uphold and balance the ethical principles of autonomy and beneficence in the medical consultation. Collaboration is the catalyst that ensures that the interaction between patient autonomy and clinician beneficence promotes patient’ health and is not reduced to ineffective or, worse still, toxic maleficence.

Considering growing evidence of the value of person-centred practice as well as its ethical imperative, training institutions have to ensure that health care students and practitioners are schooled in its precepts. There is therefore a need to identify and evaluate training interventions of person-centred practice, or at least some of the key dimensions described in this review, to both substantiate and improve student and health care practitioner learning of person-centred practice.
